# The genome sequence of the Queen of Spain Fritillary, *Issoria lathonia* (Linnaeus, 1758) (Lepidoptera: Nymphalidae)

**DOI:** 10.12688/wellcomeopenres.24702.1

**Published:** 2025-08-11

**Authors:** Yannick Chittaro, Andreas Sanchez, Camille Cornet, Kay Lucek, Charlotte J. Wright, Joana I. Meier, Mark L. Blaxter, Wellcome Sanger Institute Tree of Life Management, Samples and Laboratory team, Wellcome Sanger Institute Scientific Operations: Sequencing Operations, Wellcome Sanger Institute Tree of Life Core Informatics team, Tree of Life Core Informatics collective, Project Psyche Community

**Affiliations:** 1 Info fauna, Neuchâtel, Switzerland; 2 University of Neuchâtel, Neuchâtel, Switzerland; 3 Tree of Life, Wellcome Sanger Institute, Hinxton, England, UK

**Keywords:** Issoria lathonia, Queen of Spain Fritillary, genome sequence, chromosomal, Lepidoptera

## Abstract

We present a genome assembly from a female specimen of *Issoria lathonia* (Queen of Spain Fritillary; Arthropoda; Insecta; Lepidoptera; Nymphalidae). The assembly contains two haplotypes with total lengths of 319.19 megabases and 283.37 megabases. Most of haplotype 1 (99.92%) is scaffolded into 31 chromosomal pseudomolecules, including the W and Z sex chromosomes. Haplotype 2 was assembled to scaffold level. The mitochondrial genome has also been assembled, with a length of 15.18 kilobases.

## Species taxonomy

Eukaryota; Opisthokonta; Metazoa; Eumetazoa; Bilateria; Protostomia; Ecdysozoa; Panarthropoda; Arthropoda; Mandibulata; Pancrustacea; Hexapoda; Insecta; Dicondylia; Pterygota; Neoptera; Endopterygota; Amphiesmenoptera; Lepidoptera; Glossata; Neolepidoptera; Heteroneura; Ditrysia; Obtectomera; Papilionoidea; Nymphalidae; Heliconiinae; Argynnini; *Issoria*; *Issoria lathonia* (Linnaeus, 1758) (NCBI:txid171587)

## Background

*Issoria lathonia* colonises almost all of Europe, North Africa and the Canary Islands, and extends as far east as China ( Tshikolovets, 2011).

The species is easily distinguishable, with its angular forewings forming a concave outer margin and, above all, its large pearly spots of characteristic shape and arrangement on the underside of its hindwings. The underside of the forewing apex also features small silver spots. The upper surface is regularly speckled with round black spots. Males and females are similar. At rest, the forewings are almost entirely hidden by the hindwings.

The species thrives in a variety of environments, such as sunny edges and clearings, on embankments and roadsides, in vineyards or extensive crops, sometimes in meadows and quarries. The breeding grounds of this thermophilic species correspond mainly to the hill and mountain levels, although isolated individuals are sometimes observed above 2 400 m ( LSPN, 1987).

Eggs are laid singly under the leaves of host plants, or in their immediate vicinity, on a rock or dead leaf for example. The female prefers areas with sparse vegetation. Eggs hatch after around ten days' incubation for the spring and summer generations. Caterpillars feed almost exclusively on violets ( *Viola* spp.) ( Clarke, 2024). The imagos are generally found in sunny, flowery environments, often with large areas of bare soil, such as fallow land, natural gardens or certain ruderal areas, where they are very active foragers of numerous flowers, particularly Asteraceae and Fabaceae.

The species is plurivoltine, generally producing 2 to 3 generations a year, or even 4 in favourable years, from February to late autumn. *Issoria lathonia* is a known migrant, making partial annual migratory flights within its range ( Eitschberger & Steiniger, 1980), from populations in the south. In autumn, there is some evidence that their offspring return to the south, according to Thomas & Lewington (2014). Although the migration distances of *I. lathonia* are relatively modest compared with *V. atalanta*, for example, the butterfly is nonetheless highly mobile and can easily colonise new territories. It will sometimes establish temporary satellite populations, which will rapidly disappear if conditions become unfavourable. Large-scale population genetics based on barcode sequences suggest that *I. lathonia* is largely panmictic across Europe ( Dapporto *et al.*, 2022).

Widespread and undemanding, the species is not threatened in Europe (LC according to Van Swaay *et al*., 2010).

**Figure 1.**
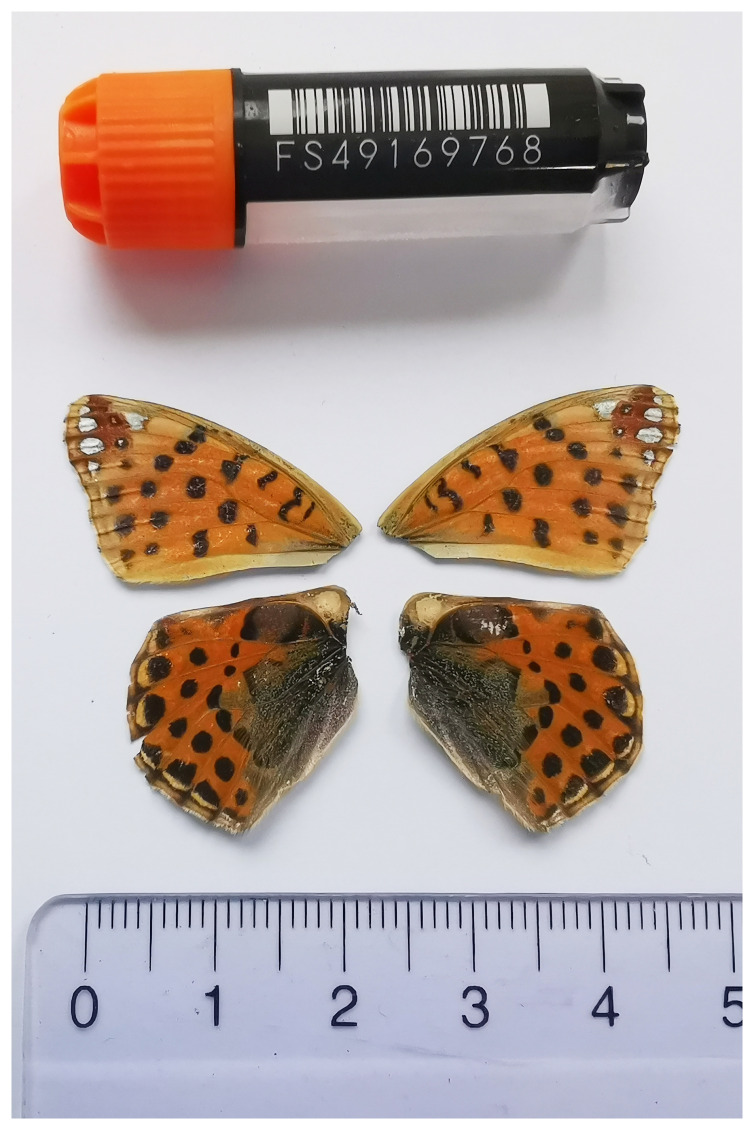
Voucher photograph of the *Issoria lathonia* (ilIssLath1) specimen used for genome sequencing.

We present a chromosome-level, haplotype-resolved genome sequence of *Issoria lathonia*, sequenced as part of Project Psyche. The sequence data was derived from a female specimen ( Figure 1) collected from Zeneggen VS, Switzerland.

## Methods

### Sample acquisition

The specimen used for genome sequencing was an adult female *Issoria lathonia* (specimen ID SAN28000129, ToLID ilIssLath1; Figure 1), collected from Zeneggen VS, Switzerland (latitude 46.2751, longitude 7.8583; elevation 1 400 m) on 26/04/2023. The specimen was collected and identified by Yannick Chittaro (Info Fauna, Neuchâtel, Switzerland).

### Nucleic acid extraction

Protocols for high molecular weight (HMW) DNA extraction developed at the Wellcome Sanger Institute (WSI) Tree of Life Core Laboratory are available on protocols.io ( Howard *et al.*, 2025). The ilIssLath1 sample was weighed and triaged to determine the appropriate extraction protocol. Tissue from the thorax was homogenised by powermashing using a PowerMasher II tissue disruptor.

HMW DNA was extracted in the WSI Scientific Operations core using the Automated MagAttract v2 protocol. DNA was sheared into an average fragment size of 12–20 kb following the Megaruptor®3 for LI PacBio protocol. Sheared DNA was purified by automated SPRI (solid-phase reversible immobilisation). The concentration of the sheared and purified DNA was assessed using a Nanodrop spectrophotometer and Qubit Fluorometer using the Qubit dsDNA High Sensitivity Assay kit. Fragment size distribution was evaluated by running the sample on the FemtoPulse system. For this sample, the final post-shearing DNA had a Qubit concentration of 48.5 ng/μL and a yield of 2 279.50 ng, with a fragment size of 15.7 kb. The 260/280 spectrophotometric ratio was 1.84, and the 260/230 ratio was 2.73.

### PacBio HiFi library preparation and sequencing

Library preparation and sequencing were performed at the WSI Scientific Operations core. Libraries were prepared using the SMRTbell Prep Kit 3.0 (Pacific Biosciences, California, USA) following the manufacturer’s instructions. The kit includes reagents for end repair/A-tailing, adapter ligation, post-ligation SMRTbell bead clean-up, and nuclease treatment. Size selection and clean-up were performed using diluted AMPure PB beads (Pacific Biosciences). DNA concentration was quantified using a Qubit Fluorometer v4.0 (ThermoFisher Scientific) and the Qubit 1X dsDNA HS assay kit. Final library fragment size was assessed with the Agilent Femto Pulse Automated Pulsed Field CE Instrument (Agilent Technologies) using the gDNA 55 kb BAC analysis kit.

The sample was sequenced on a Revio instrument (Pacific Biosciences). The prepared library was normalised to 2 nM, and 15 μL was used for making complexes. Primers were annealed and polymerases bound to generate circularised complexes, following the manufacturer’s instructions. Complexes were purified using 1.2X SMRTbell beads, then diluted to the Revio loading concentration (200–300 pM) and spiked with a Revio sequencing internal control. The sample was sequenced on a Revio 25M SMRT cell. The SMRT Link software (Pacific Biosciences), a web-based workflow manager, was used to configure and monitor the run and to carry out primary and secondary data analysis.

Specimen details, sequencing platforms, and data yields are summarised in Table 1.

**Table 1.** Specimen and sequencing data for BioProject.

Platform	PacBio HiFi	Hi-C
**ToLID**	ilIssLath1	ilIssLath1
**Specimen ID**	SAN28000129	SAN28000129
**BioSample (source individual)**	SAMEA115110011	SAMEA115110011
**BioSample (tissue)**	SAMEA115110047	SAMEA115110045
**Tissue**	thorax	head
**Sequencing platform and model**	Revio	Illumina NovaSeq X
**Run accessions**	ERR13605505	ERR13602168
**Read count total**	1.98 million	747.32 million
**Base count total**	22.30 Gb	112.85 Gb

### Hi-C

***Sample preparation and crosslinking***

The Hi-C sample was prepared from 20–50 mg of frozen head tissue of the ilIssLath1 sample using the Arima-HiC v2 kit (Arima Genomics). Following the manufacturer’s instructions, tissue was fixed and DNA crosslinked using TC buffer to a final formaldehyde concentration of 2%. The tissue was homogenised using the Diagnocine Power Masher-II. Crosslinked DNA was digested with a restriction enzyme master mix, biotinylated, and ligated. Clean-up was performed with SPRISelect beads before library preparation. DNA concentration was measured with the Qubit Fluorometer (Thermo Fisher Scientific) and Qubit HS Assay Kit. The biotinylation percentage was estimated using the Arima-HiC v2 QC beads.

***Hi-C library preparation and sequencing***

Biotinylated DNA constructs were fragmented using a Covaris E220 sonicator and size selected to 400–600 bp using SPRISelect beads. DNA was enriched with Arima-HiC v2 kit Enrichment beads. End repair, A-tailing, and adapter ligation were carried out with the NEBNext Ultra II DNA Library Prep Kit (New England Biolabs), following a modified protocol where library preparation occurs while DNA remains bound to the Enrichment beads. Library amplification was performed using KAPA HiFi HotStart mix and a custom Unique Dual Index (UDI) barcode set (Integrated DNA Technologies). Depending on sample concentration and biotinylation percentage determined at the crosslinking stage, libraries were amplified with 10–16 PCR cycles. Post-PCR clean-up was performed with SPRISelect beads. Libraries were quantified using the AccuClear Ultra High Sensitivity dsDNA Standards Assay Kit (Biotium) and a FLUOstar Omega plate reader (BMG Labtech).

Prior to sequencing, libraries were normalised to 10 ng/μL. Normalised libraries were quantified again and equimolar and/or weighted 2.8 nM pools. Pool concentrations were checked using the Agilent 4200 TapeStation (Agilent) with High Sensitivity D500 reagents before sequencing. Sequencing was performed using paired-end 150 bp reads on the Illumina NovaSeq X.

Specimen details, sequencing platforms, and data yields are summarised in Table 1.

### Genome assembly

Prior to assembly of the PacBio HiFi reads, a database of *k*-mer counts ( *k* = 31) was generated from the filtered reads using FastK. GenomeScope2 ( Ranallo-Benavidez *et al.*, 2020) was used to analyse the *k*-mer frequency distributions, providing estimates of genome size, heterozygosity, and repeat content.

The HiFi reads were assembled using Hifiasm in Hi-C phasing mode ( Cheng *et al.*, 2021; Cheng *et al.*, 2022), producing two haplotypes. Hi-C reads ( Rao *et al.*, 2014) were mapped to the primary contigs using bwa-mem2 ( Vasimuddin *et al.*, 2019). Contigs were further scaffolded with Hi-C data in YaHS ( Zhou *et al.*, 2023), using the --break option for handling potential misassemblies. The scaffolded assemblies were evaluated using Gfastats ( Formenti *et al.*, 2022), BUSCO ( Manni *et al.*, 2021) and MERQURY.FK ( Rhie *et al.*, 2020).

The mitochondrial genome was assembled using MitoHiFi ( Uliano-Silva *et al.*, 2023), which runs MitoFinder ( Allio *et al.*, 2020) and uses these annotations to select the final mitochondrial contig and to ensure the general quality of the sequence.

### Assembly curation

The assembly was decontaminated using the Assembly Screen for Cobionts and Contaminants ( ASCC) pipeline. TreeVal was used to generate the flat files and maps for use in curation. Manual curation was conducted primarily in PretextView and HiGlass ( Kerpedjiev *et al.*, 2018). Scaffolds were visually inspected and corrected as described by Howe *et al*. (2021). Manual corrections included 22 breaks and 121 joins. The curation process is documented at https://gitlab.com/wtsi-grit/rapid-curation. PretextSnapshot was used to generate a Hi-C contact map of the final assembly.

### Assembly quality assessment

Chromosomal painting was performed using lep_busco_painter using Merian elements, which represent the 32 ancestral linkage groups in Lepidoptera ( Wright *et al.*, 2024). Painting was based on gene locations from the lepidoptera_odb10 BUSCO analysis and chromosome lengths from the genome index produced using SAMtools faidx ( Danecek *et al.*, 2021). Each complete BUSCO (including both single-copy and duplicated BUSCOs) was assigned to a Merian element using a reference database, and coloured positions were plotted along chromosomes drawn to scale.

The Merqury.FK tool ( Rhie *et al.*, 2020), run in a Singularity container ( Kurtzer *et al.*, 2017), was used to evaluate *k*-mer completeness and assembly quality for both haplotypes using the *k*-mer databases ( *k* = 31) computed prior to genome assembly. The analysis outputs included assembly QV scores and completeness statistics.

The genome was analysed using the BlobToolKit pipeline, a Nextflow implementation of the earlier Snakemake BlobToolKit pipeline ( Challis *et al.*, 2020). The pipeline aligns PacBio reads using minimap2 ( Li, 2018) and SAMtools ( Danecek *et al.*, 2021) to generate coverage tracks. Simultaneously, it queries the GoaT database ( Challis *et al.*, 2023) to identify relevant BUSCO lineages and runs BUSCO ( Manni *et al.*, 2021). For the three domain-level BUSCO lineages, BUSCO genes are aligned to the UniProt Reference Proteomes database ( Bateman *et al.*, 2023) using DIAMOND blastp ( Buchfink *et al.*, 2021). The genome is divided into chunks based on the density of BUSCO genes from the closest taxonomic lineage, and each chunk is aligned to the UniProt Reference Proteomes database with DIAMOND blastx. Sequences without hits are chunked using seqtk and aligned to the NT database with blastn ( Altschul *et al.*, 1990). The BlobToolKit suite consolidates all outputs into a blobdir for visualisation. The BlobToolKit pipeline was developed using nf-core tooling ( Ewels *et al.*, 2020) and MultiQC ( Ewels *et al.*, 2016), with package management via Conda and Bioconda ( Grüning *et al.*, 2018), and containerisation through Docker ( Merkel, 2014) and Singularity ( Kurtzer *et al.*, 2017).

## Genome sequence report

### Sequence data

The genome of a specimen of *Issoria lathonia* was sequenced using Pacific Biosciences single-molecule HiFi long reads, generating 22.30 Gb (gigabases) from 1.98 million reads, which were used to assemble the genome. GenomeScope2.0 analysis estimated the haploid genome size at 295.09 Mb, with a heterozygosity of 1.94% and repeat content of 10.51%. These estimates guided expectations for the assembly. Based on the estimated genome size, the sequencing data provided approximately 73× coverage. Hi-C sequencing produced 112.85 Gb from 747.32 million reads, which were used to scaffold the assembly. Table 1 summarises the specimen and sequencing details.

### Assembly statistics

The genome was assembled into two haplotypes using Hi-C phasing. Haplotype 1 was curated to chromosome level, while haplotype 2 was assembled to scaffold level. The final assembly has a total length of 319.19 Mb in 51 scaffolds, with 161 gaps, and a scaffold N50 of 11.25 Mb ( Table 2).

**Table 2.** Genome assembly statistics.

**Assembly name**	ilIssLath1.hap1.1	ilIssLath1.hap2.1
**Assembly accession**	GCA_964270575.1	GCA_964270585.1
**Assembly level**	chromosome	scaffold
**Span (Mb)**	319.19	283.37
**Number of chromosomes**	31	N/A
**Number of contigs**	212	221
**Contig N50**	5.66 Mb	5.86 Mb
**Number of scaffolds**	51	167
**Scaffold N50**	11.25 Mb	10.87 Mb
**Longest scaffold length (Mb)**	20.18	N/A
**Sex chromosomes**	W and Z	N/A
**Organelles**	Mitochondrial genome: 15.18 kb	N/A

**Figure 2.**
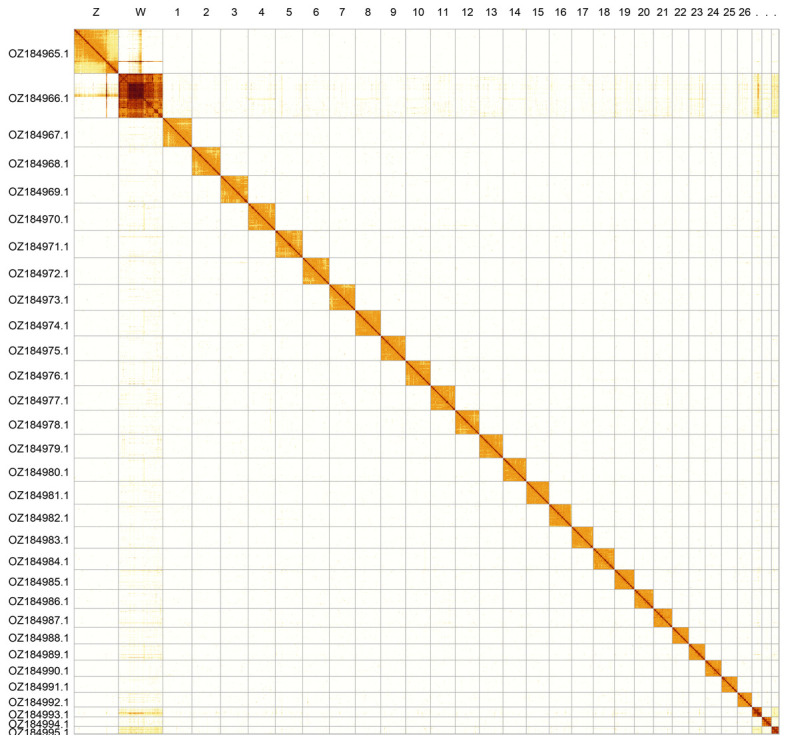
Hi-C contact map of the *Issoria lathonia* genome assembly. Assembled chromosomes are shown in order of size and labelled along the axes. The plot was generated using PretextSnapshot.

**Figure 3.**
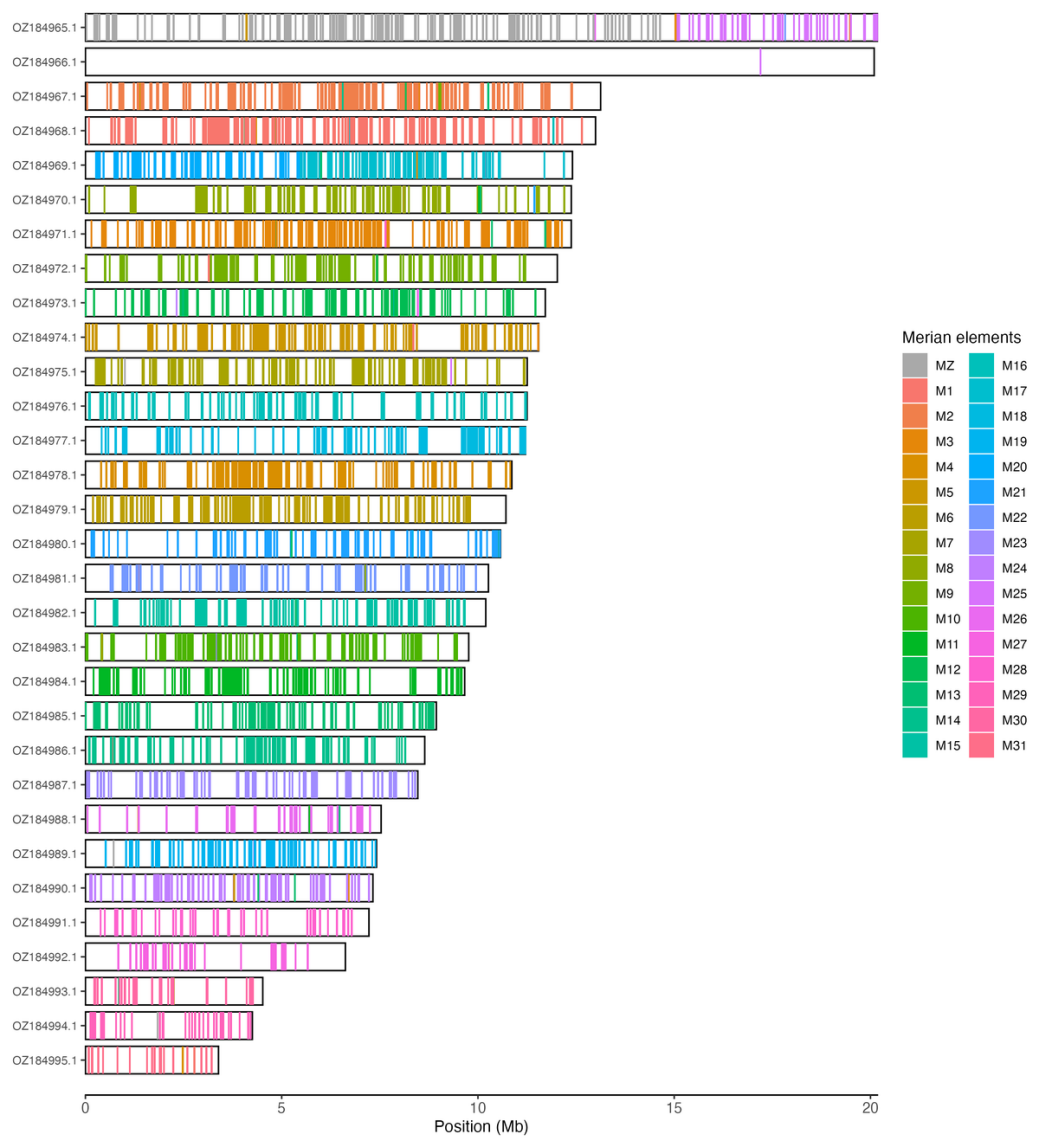
Merian elements painted across chromosomes in the ilIssLath1.hap1.1 assembly of *Issoria lathonia*. Chromosomes are drawn to scale, with the positions of orthologues shown as coloured bars. Each orthologue is coloured by the Merian element that it belongs to. All orthologues which could be assigned to Merian elements are shown.

Most of the assembly sequence (99.92%) was assigned to 31 chromosomal-level scaffolds, representing 29 autosomes and the W and Z sex chromosomes. Chromosome Z and W were assigned based on Hi-C signal. Scaffolds in chromosome W are uncertain in order and orientation. These chromosome-level scaffolds, confirmed by Hi-C data, are named according to size ( Figure 2; Table 3). Chromosome painting with Merian elements illustrates the distribution of orthologues along chromosomes and highlights patterns of chromosomal evolution relative to Lepidopteran ancestral linkage groups ( Figure 3).

**Table 3.** Chromosomal pseudomolecules in the haplotype 1 genome assembly of *Issoria lathonia* ilIssLath1.

INSDC accession	Molecule	Length (Mb)	GC%	Assigned Merian elements
OZ184967.1	1	13.12	31	M2
OZ184968.1	2	13	31.50	M1
OZ184969.1	3	12.41	31	M17;M20
OZ184970.1	4	12.38	31	M8
OZ184971.1	5	12.38	31.50	M3
OZ184972.1	6	12.02	30.50	M9
OZ184973.1	7	11.72	30.50	M12
OZ184974.1	8	11.55	31	M5
OZ184975.1	9	11.26	31	M7
OZ184976.1	10	11.25	30.50	M16
OZ184977.1	11	11.21	30.50	M18
OZ184978.1	12	10.86	31	M4
OZ184979.1	13	10.71	31	M6
OZ184980.1	14	10.57	31	M21
OZ184981.1	15	10.27	30.50	M22
OZ184982.1	16	10.20	31	M15
OZ184983.1	17	9.76	31	M10
OZ184984.1	18	9.66	31.50	M11
OZ184985.1	19	8.94	30.50	M13
OZ184986.1	20	8.64	31	M14
OZ184987.1	21	8.47	31	M23
OZ184988.1	22	7.53	30.50	M26
OZ184989.1	23	7.42	31.50	M19
OZ184990.1	24	7.32	30.50	M24
OZ184991.1	25	7.22	31	M28
OZ184992.1	26	6.62	30.50	M27
OZ184993.1	27	4.52	34	M30
OZ184994.1	28	4.25	31.50	M29
OZ184995.1	29	3.39	32.50	M31
OZ184966.1	W	20.09	38.50	N/A
OZ184965.1	Z	20.18	31.50	M25;MZ

The mitochondrial genome was also assembled. This sequence is included as a contig in the multifasta file of the genome submission and as a standalone record.

### Assembly quality metrics

**Figure 4.**
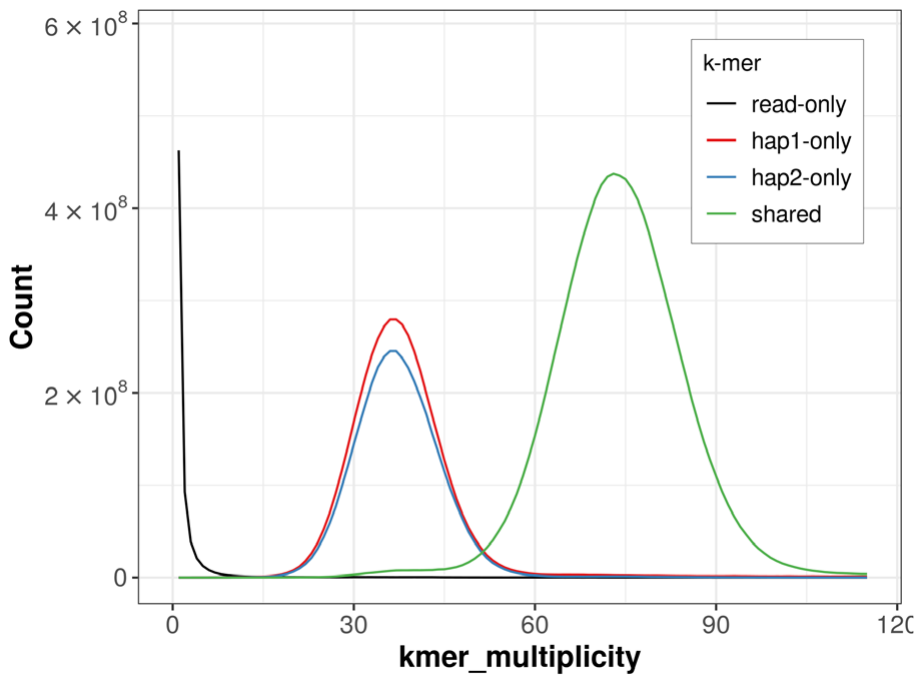
Evaluation of *k*-mer completeness using MerquryFK. This plot illustrates the recovery of *k*-mers from the original read data in the final assemblies. The horizontal axis represents *k*-mer multiplicity, and the vertical axis shows the number of *k*-mers. The black curve represents *k*-mers that appear in the reads but are not assembled. The green curve (the homozygous peak) corresponds to *k*-mers shared by both haplotypes and the red and blue curves (the heterozygous peaks) show *k*-mers found only in one of the haplotypes.

**Figure 5.**
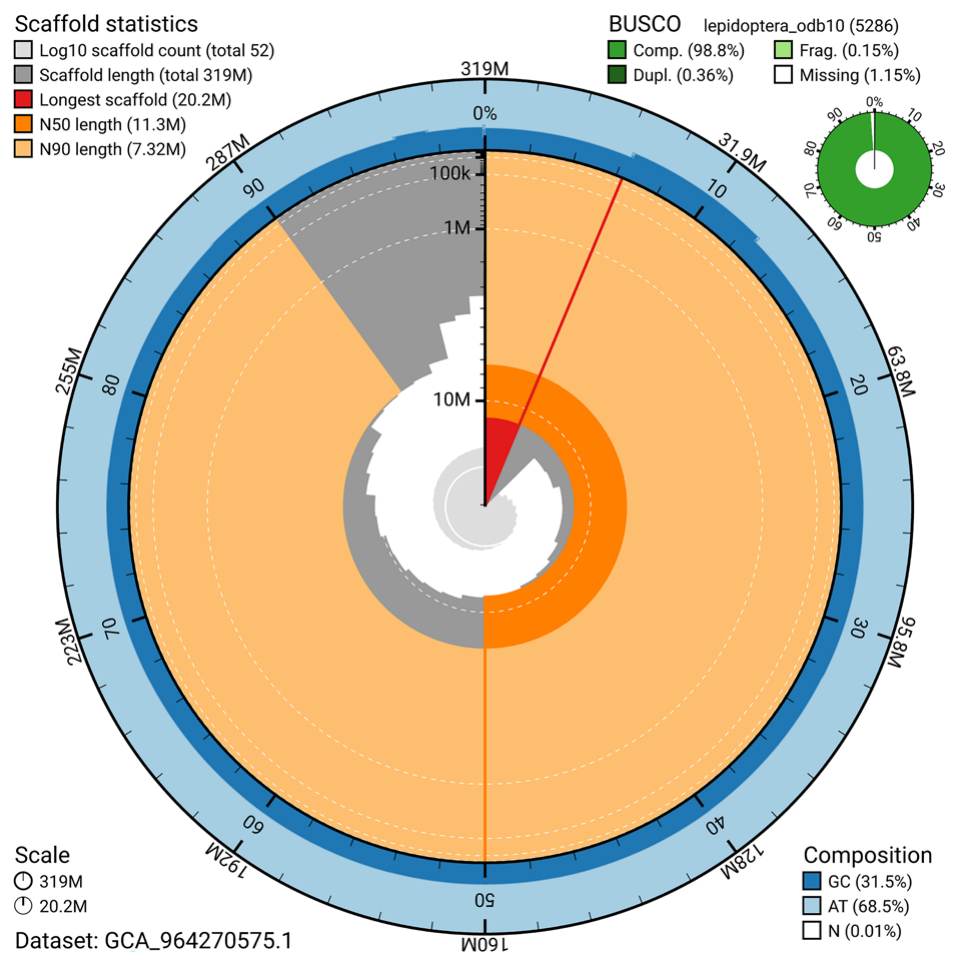
Assembly metrics for ilIssLath1.hap1.1. The BlobToolKit snail plot provides an overview of assembly metrics and BUSCO gene completeness. The circumference represents the length of the whole genome sequence, and the main plot is divided into 1,000 bins around the circumference. The outermost blue tracks display the distribution of GC, AT, and N percentages across the bins. Scaffolds are arranged clockwise from longest to shortest and are depicted in dark grey. The longest scaffold is indicated by the red arc, and the deeper orange and pale orange arcs represent the N50 and N90 lengths. A light grey spiral at the centre shows the cumulative scaffold count on a logarithmic scale. A summary of complete, fragmented, duplicated, and missing BUSCO genes in the set is presented at the top right. An interactive version of this figure can be accessed on the BlobToolKit viewer.

**Figure 6.**
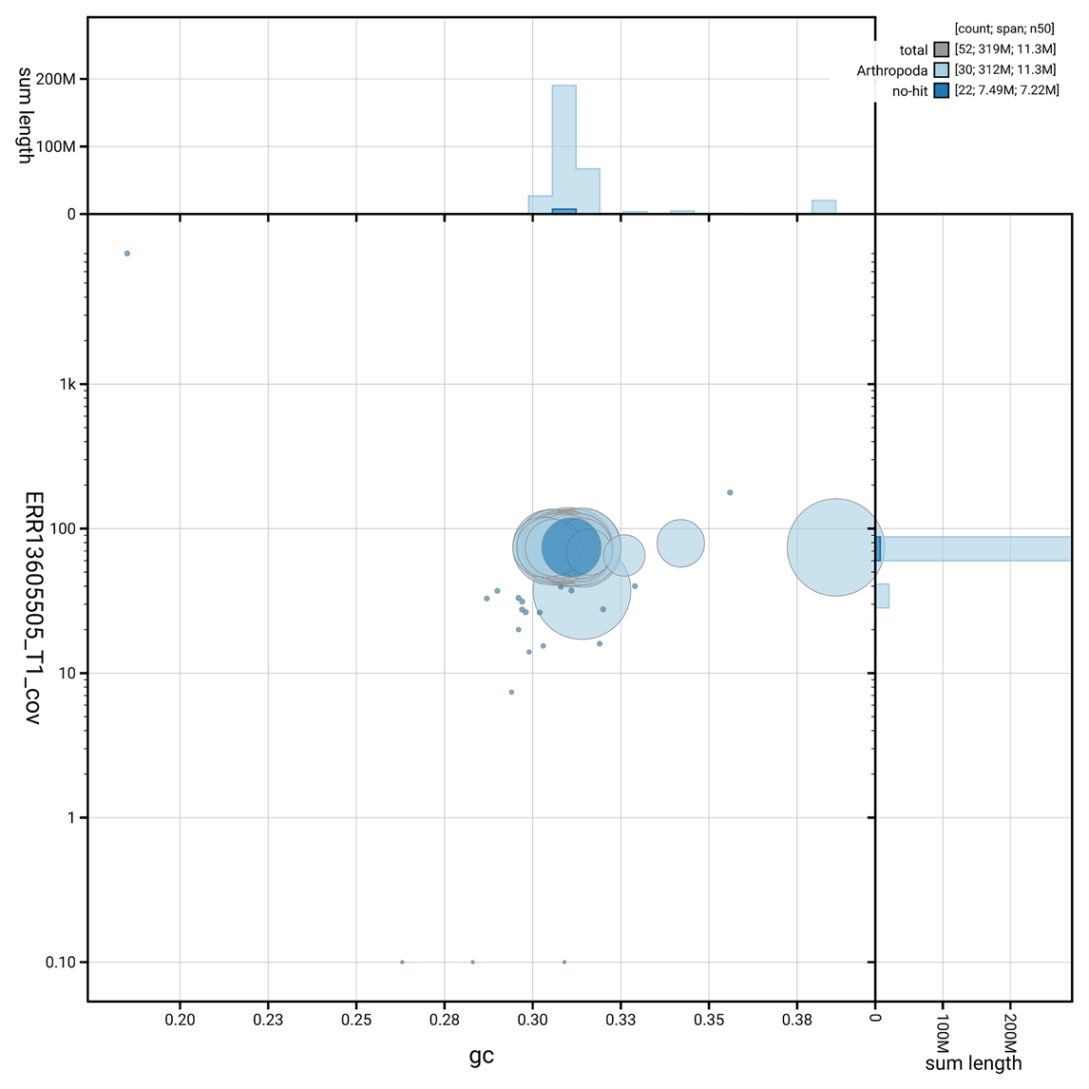
BlobToolKit GC-coverage plot for ilIssLath1.hap1.1. Blob plot showing sequence coverage (vertical axis) and GC content (horizontal axis). The circles represent scaffolds, with the size proportional to scaffold length and the colour representing phylum membership. The histograms along the axes display the total length of sequences distributed across different levels of coverage and GC content. An interactive version of this figure is available on the BlobToolKit viewer.

For haplotype 1, the estimated QV is 55.7, and for haplotype 2, 55.8. When the two haplotypes are combined, the assembly achieves an estimated QV of 55.7. The *k*-mer completeness is 71.32% for haplotype 1, 66.68% for haplotype 2, and 99.84% for the combined haplotypes ( Figure 4). BUSCO analysis using the lepidoptera_odb10 reference set ( *n* = 5 286) ( Kriventseva *et al.*, 2019) identified 98.8% of the expected gene set (single = 98.5%, duplicated = 0.4%) for haplotype 1. The snail plot in Figure 5 summarises the scaffold length distribution and other assembly statistics for haplotype 1. The blob plot in Figure 6 shows the distribution of scaffolds by GC proportion and coverage for haplotype 1.

Table 4 lists the assembly metric benchmarks adapted from Rhie *et al.* (2021) the Earth BioGenome Project Report on Assembly Standards September 2024. The EBP metric, calculated for the haplotype 1, is **6.C.Q55**, meeting the recommended reference standard.

**Table 4.** Earth Biogenome Project summary metrics for the *Issoria lathonia* assembly.

Measure	Value	Benchmark
EBP summary (haplotype 1)	6.7.Q55	6.C.Q40
Contig N50 length	5.66 Mb	≥ 1 Mb
Scaffold N50 length	11.25 Mb	= chromosome N50
Consensus quality (QV)	Haplotype 1: 55.7; haplotype 2: 55.8; combined: 55.7	≥ 40
*k*-mer completeness	Haplotype 1: 71.32%; Haplotype 2: 66.68%; combined: 99.84%	≥ 95%
BUSCO	C:98.8%[S:98.5%‚D:0.4%]‚ F:0.2%‚M:1.0%‚n:5 286	S > 90%; D < 5%
Percentage of assembly assigned to chromosomes	99.92%	≥ 90%

### Wellcome Sanger Institute – Legal and Governance

The materials that have contributed to this genome note have been supplied by a Tree of Life collaborator. The Wellcome Sanger Institute employs a process whereby due diligence is carried out proportionate to the nature of the materials themselves, and the circumstances under which they have been/are to be collected and provided for use. The purpose of this is to address and mitigate any potential legal and/or ethical implications of receipt and use of the materials as part of the research project, and to ensure that in doing so, we align with best practice wherever possible. The overarching areas of consideration are:

- Ethical review of provenance and sourcing of the material
- Legality of collection, transfer and use (national and international).

Each transfer of samples is undertaken according to a Research Collaboration Agreement or Material Transfer Agreement entered into by the Tree of Life collaborator, Genome Research Limited (operating as the Wellcome Sanger Institute), and in some circumstances, other Tree of Life collaborators.

## Data Availability

European Nucleotide Archive: Issoria lathonia (Queen of Spain fritillary). Accession number PRJEB79184. The genome sequence is released openly for reuse. The *Issoria lathonia* genome sequencing initiative is part of the Sanger Institute Tree of Life Programme (PRJEB43745) and Project Psyche (PRJEB71705). All raw sequence data and the assembly have been deposited in INSDC databases. The genome will be annotated using available RNA-Seq data and presented through Ensembl at the European Bioinformatics Institute. Raw data and assembly accession identifiers are reported in Table 1 and Table 2. Pipelines used for genome assembly at the WSI Tree of Life are available at https://pipelines.tol.sanger.ac.uk/pipelines. Table 5 lists software versions used in this study.

## Author information

Contributors are listed at the following links:

- Wellcome Sanger Institute Tree of Life Management, Samples and Laboratory team
- Wellcome Sanger Institute Scientific Operations – Sequencing Operations
- Wellcome Sanger Institute Tree of Life Core Informatics team
- Tree of Life Core Informatics collective
- Project Psyche Community.
