# Supplementary material for: The genome sequence of the Queen of Spain Fritillary, Issoria lathonia (Linnaeus, 1758) (Lepidoptera: Nymphalidae)
Source: Wellcome Open Res. 2025 Aug 11;10:427. [Version 1] doi: 10.12688/wellcomeopenres.24702.1 (PMC12457893; doi:10.12688/wellcomeopenres.24702.1)
Supplement: Peer review history

# Reviewer report: Reviewer response for version 1

**DOI:** 10.21956/wellcomeopenres.27218.r129772

**Published:** 2025-09-23

**Authors:** Mukta Joshi

**Affiliations:** 1 University of Oulu, Oulu, Finland

The manuscript presents the genome of Queen of Spain Fritillary butterfly, *Issoria lathonia* produced by the Tree of Life consortium (Wellcome Sanger Institute), as part of Project Psyche. Project Psyche is an important initiative that is helping create an exceptional resource for the global Lepidoptera research community (both genomes and the standardisation of several routine and advanced analyses performed as part of the genome assembly process). Manuscript contains good details of standard genome assembly protocols including HMW DNA extraction, PacBio HiFi library prep, Hi-C library prep, assembly and curation, as well as detailed quality assessment (which I have not seen in many of the previous genome notes). The data generated is of high quality, serving as a valuable addition to molecular resources currently available for Nymphalids. Some informative analyses are done (such as chromosome painting using Merian elements) which could potentially be helpful for researchers studying patterns of chromosomal evolution in this group.

Are sufficient details of methods and materials provided to allow replication by others?

Yes

Is the rationale for creating the dataset(s) clearly described?

Yes

Are the datasets clearly presented in a useable and accessible format?

Yes

Are the protocols appropriate and is the work technically sound?

Yes

Reviewer Expertise:

Evolutionary Biology, Molecular Systematics, Molecular Evolution, Speciation

I confirm that I have read this submission and believe that I have an appropriate level of expertise to confirm that it is of an acceptable scientific standard.

# Reviewer report: Reviewer response for version 1

**DOI:** 10.21956/wellcomeopenres.27218.r130808

**Published:** 2025-09-23

**Authors:** Emily C Giles

**Affiliations:** 1 Universidad Austral de Chile, Valdivia, Los Ríos Region, Chile

The authors present a haplotype resolved chromosome level assembly for a butterfly from the family Nymphalidae. Most of haplotype 1 falls into 31 pseudo chromosomes. A mitogenome assembly is also presented.

 The introduction gives basic biology of the species. The authors describe the species distribution, reproduction, and later go on to describe its migratory patterns. While this information is very pertinent to genetic and genomic studies in this group, the description of the species’ distribution and migration is northern-centric. The authors assume the connection between autumn - southward migration. I had to reread the initial part of the Introduction to verify that the distribution of the species is restricted to the Northern hemisphere. The authors should make this more explicit or describe migration in terms of high latitude - low latitude. Furthermore, comparison of migration distance between species of this genus are not relevant for a wider audience. What does “modest compared to V. atalanta” refer to? What does “undemanding” refer to? The narrow-scope of the introduction should be improved.

 Based on the estimated genome size, sequencing produced 73X coverage. An extremely robust decontamination protocol was used, and the quality of the genome is extremely high with high complete BUSCOs. I have no concerns with the assembly, which appears to be of great quality and completeness.

Are sufficient details of methods and materials provided to allow replication by others?

Yes

Is the rationale for creating the dataset(s) clearly described?

Partly

Are the datasets clearly presented in a useable and accessible format?

Yes

Are the protocols appropriate and is the work technically sound?

Yes

Reviewer Expertise:

Comparative genomics, population genomics, speciation, marine genomics, biosecurity

I confirm that I have read this submission and believe that I have an appropriate level of expertise to confirm that it is of an acceptable scientific standard.
